# Application of Augmented Reality in Reverse Total Shoulder Arthroplasty: A Systematic Review

**DOI:** 10.3390/jcm14155533

**Published:** 2025-08-06

**Authors:** Jan Orlewski, Bettina Hochreiter, Karl Wieser, Philipp Kriechling

**Affiliations:** Department of Orthopaedics, Balgrist University Hospital, University of Zurich, Forchstrasse 340, 8008 Zurich, Switzerland

**Keywords:** reverse total shoulder arthroplasty, systematic review, augmented reality, immersive techniques

## Abstract

**Background:** Reverse total shoulder arthroplasty (RTSA) is increasingly used for managing cuff tear arthropathy, osteoarthritis, complex fractures, and revision procedures. As the demand for surgical precision and reproducibility grows, immersive technologies such as virtual reality (VR), augmented reality (AR), and metaverse-based platforms are being explored for surgical training, intraoperative guidance, and rehabilitation. While early data suggest potential benefits, a focused synthesis specific to RTSA is lacking. **Methods:** This systematic review was conducted in accordance with PRISMA 2020 guidelines. A comprehensive search of PubMed, Scopus, and Cochrane Library databases was performed through 30 May 2025. Eligible studies included those evaluating immersive technologies in the context of RTSA for skill acquisition or intraoperative guidance. Only peer-reviewed articles published in English were included. Data were synthesized narratively due to heterogeneity in study design and outcome metrics. **Results:** Out of 628 records screened, 21 studies met the inclusion criteria. Five studies evaluated immersive VR for surgical training: four randomized controlled trials and one retrospective case series. VR training improved procedural efficiency and showed non-inferiority to cadaveric training. Sixteen studies investigated intraoperative navigation or AR guidance. Clinical and cadaveric studies consistently reported improved accuracy in glenoid baseplate positioning with reduced angular and linear deviations in postoperative controls as compared to preoperative planning. **Conclusions:** Immersive technologies show promise in enhancing training, intraoperative accuracy, and procedural consistency in RTSA. VR and AR platforms may support standardized surgical education and precision-based practice, but their broad clinical impact remains limited by small sample sizes, heterogeneous methodologies, and limited long-term outcomes. Further multicenter trials with standardized endpoints and cost-effectiveness analyses are warranted. Postoperative rehabilitation using immersive technologies in RTSA remains underexplored and presents an opportunity for future research.

## 1. Introduction

Reverse total shoulder arthroplasty (RTSA) has become an essential treatment modality for patients with cuff tear arthropathy [1,2], osteoarthritis [3], complex fractures [4], or revision arthroplasty [5]. As the number of procedures continues to rise globally [6,7], so does the demand for improved reproducibility, efficiency, and surgical precision in education, operative technique, and postoperative rehabilitation. Emerging immersive technologies, including virtual reality (VR), augmented reality (AR), mixed reality (MR), and, more recently, concepts from the broader “metaverse”, are being increasingly explored as tools to enhance these outcomes [8]. Although all above mentioned are considered immersive technologies, they differ in how they blend the digital and physical worlds. AR enhances the perception of the real world by overlaying digital content onto it. VR creates a fully simulated digital environment, essentially replacing the real world. MR combines elements of both AR and VR, allowing for interaction between digital and physical objects.

The use of immersive technologies in shoulder arthroplasty has gained traction, particularly for RTSA, where glenoid exposure and implant positioning, even considering modern navigation techniques, are technically demanding [9,10]. VR and AR offer interactive environments that can replicate complex surgical tasks, anatomical structures, and decision-making scenarios. These technologies have shown promise in three main domains relevant to RTSA: surgical training and skill acquisition [11], intraoperative visualization and guidance [12], and rehabilitation [13]. In each area, immersive technologies may contribute to standardization, improved learning curves, and potentially enhanced patient outcomes—though the degree to which this promise has translated into clinical practice remains uncertain.

While prior reviews, such as Longo et al. [14], have synthesized early evidence on immersive technologies in shoulder arthroplasty, a comprehensive and critical synthesis focused specifically on RTSA—and including the distinct roles of VR, AR, and the metaverse across the full surgical spectrum—has not yet been conducted. Moreover, the literature remains heterogeneous in terms of study design, intervention types, and outcome metrics, necessitating a structured and transparent review methodology.

The objective of this systematic review was to evaluate the current evidence regarding the use of immersive technologies (VR, AR, and metaverse applications) in RTSA. Specifically, the authors examined their roles in skill acquisition and training as well as intraoperative navigation. This review adhered to PRISMA guidelines [15] and aims to critically appraise methodological quality, identify gaps in clinical validation, and suggest strategic priorities for translational research and implementation. It was hypothesized that immersive technologies can improve surgical training and achieve reliable RTSA implant positioning.

## 2. Methods

### 2.1. Study Design and Protocol

This systematic review was conducted in accordance with the Preferred Reporting Items for Systematic Reviews and Meta-Analyses (PRISMA) 2020 guidelines [15]. The review protocol was designed to evaluate the use of immersive technologies, including VR, AR, and metaverse-based platforms, in the context of RTSA. The current state of literature considers their application in surgical training and skill acquisition (understood as processes leading to learning or mastering a certain surgical skill) as well as intraoperative planning or navigation (considering their use in aiding component placement during RTSA procedures). Reviewed studies were assigned to one of the two main groups based on the researched topic. Information on the use in postoperative rehabilitation has also been gathered. The study was not registered in any register. In our country (Switzerland), systematic reviews are exempt from IRB (Institutional Review Board) approval; therefore, no such approval was obtained. A review protocol was not prepared. The study received no funding. The authors have no conflict of interest to declare related to the study.

### 2.2. Eligibility Criteria

Studies were included if they investigated the use of VR, AR, or metaverse technologies in the setting of RTSA implantation; focused on any of the following domains: training and education, intraoperative navigation or guidance, or rehabilitation; and were peer-reviewed, published in English, and provided extractable data relevant to the review objectives. The team excluded studies that did not differentiate RTSA from other types of shoulder arthroplasty (i.e., anatomical total shoulder arthroplasty or hemiarthroplasty); publications in foreign languages other than mentioned above, proceeding papers, book chapters, book reviews, meeting abstracts, theses, interviews, and editorial materials.

### 2.3. Search Strategy and Study Selection

A comprehensive literature search was performed using PubMed, Scopus, and the Cochrane Library until 30 May 2025. Search terms included keywords such as: ((virtual reality) OR (VR) OR (augmented reality) OR (AR) OR (metaverse) OR (MR) OR (mixed reality)) AND ((shoulder arthroplasty) OR (reverse shoulder replacement) OR (RTSA) OR (RSA)). References of included articles were manually screened to identify additional relevant studies. All retrieved records were imported into a reference manager, and duplicates were removed. Two reviewers (J.O., P.K.) independently screened titles and abstracts for relevance. Full-text articles were then reviewed based on the eligibility criteria. Discrepancies were resolved through discussion and mutual agreement. In case of disagreement, a third reviewer (B.H. or K.W.) was consulted for consensus.

### 2.4. Data Extraction

Data were independently extracted by two reviewers using a standardized form. Extracted fields included the following:

- Study characteristics (author, year, study design, number of participants)
- Application setting (training and skill acquiring or intraoperative guiding and navigation)
- Comparison group (if any)
- Outcome measures and key findings such as baseplate position (entry point at glenoid, version, inclination, medialization, outliers) and surgical time

### 2.5. Data Synthesis

Due to the heterogeneity in study designs, intervention types, and outcome measures, a narrative synthesis was conducted. Studies were grouped into two thematic categories:

1. Skill acquisition and training
2. Intraoperative planning and navigation

Quantitative synthesis (meta-analysis) was not performed due to variability in metrics and endpoints. The topic of immersive technologies in postoperative rehabilitation was not included in the results section, as the authors found no studies meeting the inclusion criteria. Risk of bias for randomized trials was assessed using the RoB 2 tool established by Cochrane [16].

## 3. Results

**Figure 1:**
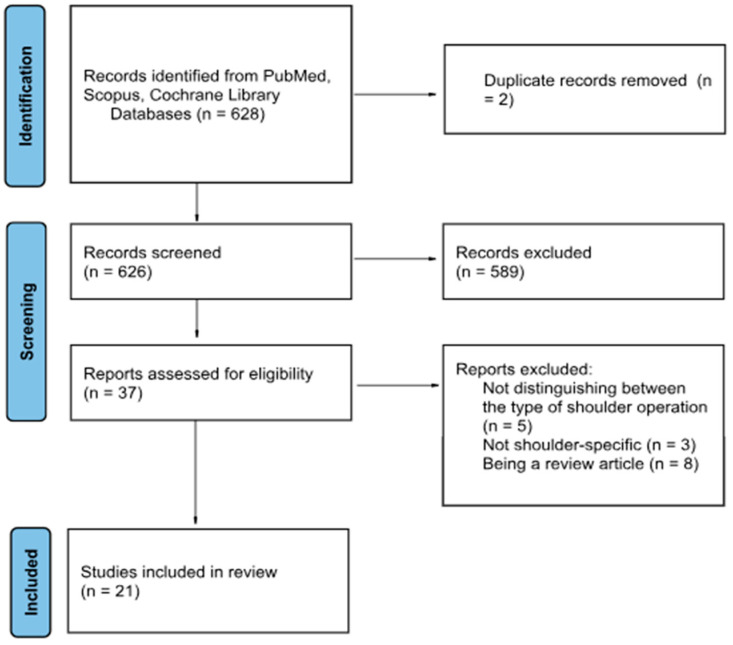
PRISMA flow-chart of included studies.

Overall, a total of 21 studies were included (Figure 1). Of those, five investigated immersive technologies for training, and sixteen investigated the application in intraoperative navigation or guidance. Only one article evaluated the intraoperative clinical use as a navigation device. However, no relevant study regarding postoperative rehabilitation was found.

### 3.1. Training and Skill Acquisition

**Figure 2:**
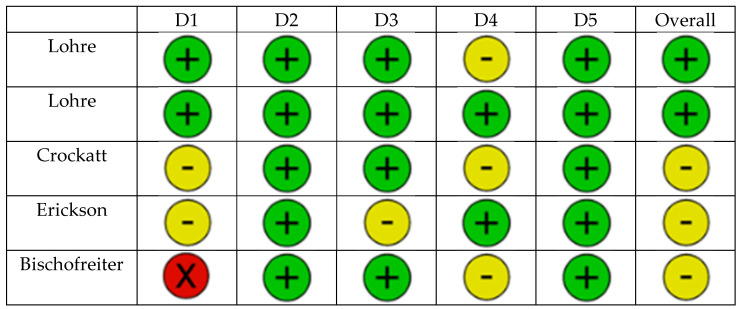
The diagram presents the risk of bias for the educational studies in accordance to the difference domains (Domain 1 (**D1**)—Bias arising from the randomization process, Domain 2 (**D2**)—Bias due to deviations from intended interventions, Domain 3 (**D3**)—Bias due to missing outcome data, Domain 4 (**D4**)—Bias in measurement of the outcome, Domain 5 (**D5**)—Bias in selection of the reported result). The risk of bias was assessed as low risk (green), some concerns (yellow), and high risk (red) [17,18,19,20,21].

A total of five studies explored immersive VR as a training tool for RTSA implantation (Table 1, Figure 2). Four were randomized controlled trials comparing immersive VR platforms to conventional training methods such as cadaver labs or technical literature. The fifth study was a retrospective case series investigating performance over time using an AR–guided system in a clinical setting.

Lohre et al. [17] conducted an RCT comparing immersive VR training to traditional text-based learning for glenoid exposure. Orthopedic residents who used the PrecisionOS VR platform were quicker to complete their training sessions (mean 4.1 ± 2.5 min vs. 16.1 ± 2.6 min, *p* < 0.001) and improvement of cumulative objective structured assessments of technical skills (OSATS) scores (mean 15.9 ± 2.5 vs. 9.4 ± 3.2, *p* < 0.001). Residents in the VR group completed the cadaveric procedure faster than those in the control group (mean 17.1 ± 5.7 min vs. 25.3 ± 32.5 min), although the difference was not statistically significant (*p* = 0.13). The study concluded that immersive VR significantly improved technical skill acquisition and validated its transferability to a clinical setting.

In a subsequent trial, Lohre et al. [18] evaluated immersive VR against using a technical journal article as a control in senior orthopedic residents and consultants. The VR group achieved significantly higher OSATS scores for instrument handling (mean 3.25 vs. 3.0, *p* = 0.03), and completed the cadaveric glenoid exposure task faster (mean time 14 ± 7 vs. 21 ± 6 min, *p* = 0.04). The VR system was significantly more efficient for resident and expert surgeons than traditional teaching.

Crockatt et al. [19] directly compared immersive VR training with cadaveric labs for junior orthopedic residents learning glenoid baseplate implantation. Performance outcomes (OSATS and completion time) showed no statistically significant differences between groups, suggesting that VR was non-inferior to cadaveric training in skill acquisition. The study also highlighted the cost and logistical advantages of VR simulation.

Erickson et al. [20] investigated the ability of orthopedic residents to place the central glenoid guide wire into Walch B2 glenoid models, comparing 30 procedures with standard freehand technique to 30 procedures with mixed VR guidance. VR training included 10–15 min of training before the procedure and the display of the hologram during pin placement. The difference from the plan for the entry point reached 2.17 ± 0.93 mm and 1.66 ± 0.70 mm for freehand and MR, respectively. The values for deviation from the planned version were 11.69 ± 8.02° (freehand) and 5.3 ± 3.57° (MR), and for version 8.4 ± 6.68° and 7.11 ± 4.96°.

Bischofreiter et al. [21] presented a retrospective analysis of the learning curve of a single high-volume shoulder surgeon using the AR-based NextAR system. Over 20 RTSA procedures, significant improvements were seen in surgical time (reduction from 137.2 ± 15.3 to 91.4 ± 11.7 min, *p* < 0.001) and intraoperative blood loss (reduction from 868.4 ± 151.1 mL to 474 ± 257.2 mL, *p* = 0.005).

### 3.2. Intraoperative Navigation and Guiding

**Figure 3:**
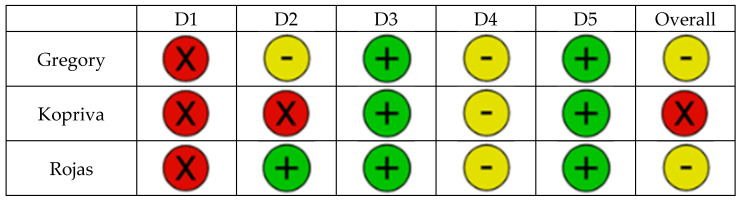
The diagram presents the risk of bias for the clinical studies in accordance to the difference domains (Domain 1 (**D1**)—Bias arising from the randomization process, Domain 2 (**D2**)—Bias due to deviations from intended interventions, Domain 3 (**D3**)—Bias due to missing outcome data, Domain 4 (**D4**)—Bias in measurement of the outcome, Domain 5 (**D5**)—Bias in selection of the reported result). The risk of bias was assessed as low risk (green), some concerns (yellow), and high risk (red) [22,23,24].

**Figure 4:**
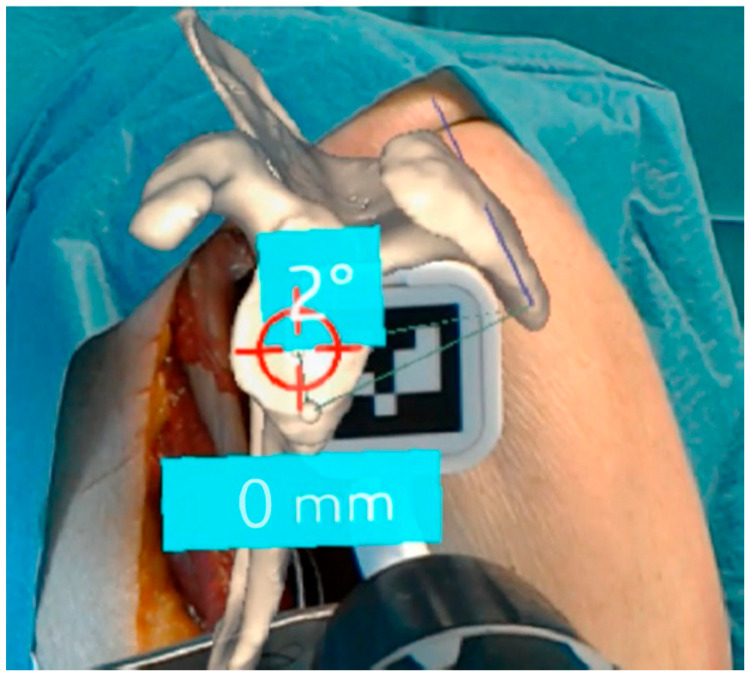
Illustration of intraoperative use of augmented reality for navigation in a cadaveric study. A hologram was overlaid to the real anatomy to show the surgeon where the scapula was. A tracking marker was attached to the aiming guide for later pine placement. The numbers showed the 3D angulation of the entry wire and the distance to the entry point in mm to achieve the planned position [25].

**Figure 5:**
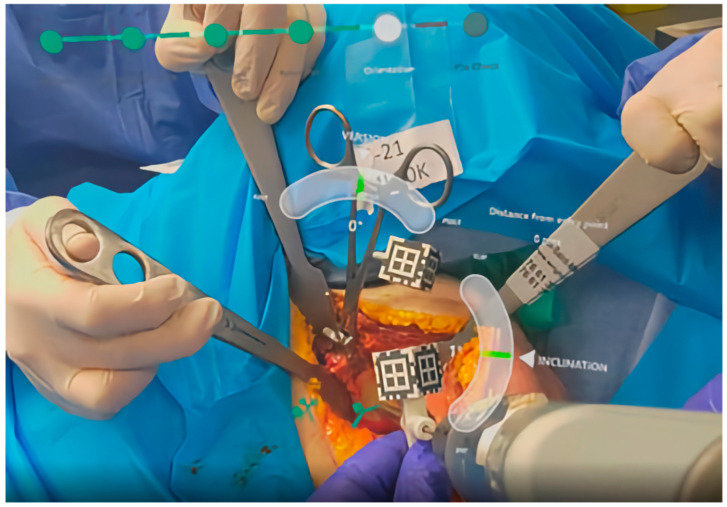
Illustration of intraoperative use of augmented reality for navigation in a cadaveric study. The shoulder was incised through a deltopectoral approach, the glenoid was presented by use of retractors. One 3D marker was placed at the coracoid process using a specific clamp, another marker was attached to the aiming guide for central pin placement. The illustration further shows placement of the central guidewire for later implantation of the baseplate [27].

Sixteen studies investigated immersive technologies to aid component placement in RTSA. These included three clinical trials [22,23,24] (Figure 3), five cadaveric simulations [25,26,27,28,29], and eight technical feasibility reports [30,31,32,33,34,35,36,37] using models such as saw bones. Most studies focused on AR-enhanced navigation, using CT-based planning data to be optically overlaid during surgery through head-mounted displays or smart glasses (Figure 4 and Figure 5). The included devices were AR head-mounted displays (e.g., Microsoft HoloLens, Pixee Medical, NextAR). Figure 4 depicts the superimposed hologram of the scapula, as seen through the head-mounted display in a cadaveric model, with the deviation in millimeters and in degrees from the planned entry point and trajectory of the guidewire. Similarly, Figure 5 shows the surgeon’s perspective in a cadaveric model, with real-time deviation from the planned position and orientation of the guide pin.

The main outcomes that were evaluated across these studies comprised glenoid baseplate positioning accuracy (version, inclination, entry point), surgical workflow and setup efficiency, clinical outcomes, and complication rates.

Berhouet et al. [30], Kriechling et al. [31], Schlueter-Brust et al. [32], Gu et al. [35], Trehin et al. [36], Fleet et al. [33], Italia et al. [34], and Abdic et al. [37] evaluated the feasibility using non-cadaveric models. Overall, the authors demonstrated an acceptable deviation from the surgical plan with regard to entry point, version, and inclination (Table 2, Table 3 and Table 4).

Another five studies tested the subsequent application in cadaveric models using the MS HoloLens 1 or 2, as well as the Pixee Next AR system. The detailed results of those studies are shown in Table 4. All studies achieved comparable results for the entry point between 1 mm and 3 mm deviation from the plan. Analysis from the plan for version and inclination similarly revealed low values between 0.7° and 3.8° of deviation. While Kriechling et al. [25], Rojas et al. [26], Sanchez-Sotelo et al. [27], and Dordain et al. [28] compared the deviation from the surgical plan, Dey Hazra [29] conducted a comparative study to differentiate between the freehand technique and the use of immersive technologies. The authors described a reduction in deviation with the use of AR navigation. However, the mean deviation between planned and achieved vectors was comparable between both techniques (Table 2 and Table 4).

Analysis of the application in the real clinical scenario revealed only three relevant studies (Table 3 and Table 4), of which two used the technology to visualize the plan intraoperatively. Gregory et al. [22] conducted a multicenter study that brought multiple shoulder surgeons together while one center was performing the operation. Kopriva et al. [23] published the use of AR as a visualization intraoperatively, but without navigation. Only the study by Rojas et al. [24] used AR as an in vivo navigation tool, revealing acceptable deviation rates from the plan.

## 4. Discussion

This systematic review provides a comprehensive analysis of the current evidence regarding the use of immersive technologies to facilitate RTSA implantation. With respect to both surgical training and intraoperative navigation, those modern systems were shown to effectively and consistently enhance technical skill acquisition and improve implant placement accuracy in both clinical and cadaveric settings.

### 4.1. Orthopedic Training

The integration of immersive technologies into orthopedic surgical education has shown particular promise in shoulder arthroplasty, where the complexity of glenoid exposure and implant placement poses consistent challenges to trainees. The five studies included in this review [17,18,19,20,21] offered compelling early evidence that immersive VR and AR platforms can significantly improve training efficiency, technical competence, and procedural familiarity in RTSA.

The randomized controlled trials by Lohre et al. [17,18] provided a strong evidence base. Their findings consistently demonstrated that VR-trained orthopedic residents outperform peers trained with traditional passive learning tools (e.g., surgical videos, technical literature) across metrics including OSATS scores, procedural time, and knowledge retention. Notably, the studies validated the transferability of VR-acquired skills to cadaveric simulations—an important bridge toward operative performance. VR systems could have implications for scaling surgical education globally.

The comparison of VR simulation to cadaveric lab training by Crockatt et al. [19] offered further insight. Although no significant differences in performance were found, the study confirmed that immersive VR was non-inferior to cadaveric education. This is particularly meaningful in institutions where access to cadaveric resources is limited by cost, ethical constraints, or logistical barriers.

The case series by Bischofreiter et al. [21] shifts focus to the applicability of AR-guided platforms in a real-world operative setting. Although the study does not address early training directly, it demonstrated that immersive guidance systems could have a quick learning curve. Over the course of 20 consecutive procedures, a substantial reduction in operative time and intraoperative blood loss was observed, suggesting that immersive guidance tools may offer value across experience levels—not only for novice training.

Despite these positive findings, several limitations warrant caution. All the aforementioned studies have been conducted in single-center or simulated environments. While two studies confirmed skill transfer to cadaveric models, none directly assessed long-term translation to intraoperative performance. Sample sizes were relatively small, and most studies relied on immediate post-training assessments rather than longitudinal tracking. Additionally, the identified studies used a single, particular VR platform, limiting the generalizability of results across other systems. Future research in this topic should prioritize multicenter trials with standardized assessment frameworks and long-term follow-up. Comparative studies between different VR/AR systems may also clarify platform-specific strengths and weaknesses. Finally, efforts to integrate immersive modules into residency curricula and certification frameworks should be explored to better define their role within formal surgical education.

### 4.2. Clinical Application

The growing adoption of immersive technologies in RTSA also reflects a broader shift toward digital precision in orthopedic surgery. Across 16 cadaveric and clinical studies, AR-based systems consistently demonstrated improvements in glenoid component placement accuracy and surgical reproducibility.

The findings reinforce the relevance of intraoperative guidance tools in achieving angular precision during glenoid baseplate implantation. Of the analyzed studies, eight were conducted as feasibility studies using scapula models, five were performed in a cadaveric setting, and three studies evaluated the intraoperative, clinical usability in real patient scenarios. The satisfactory results of the feasibility studies could reliably be transferred into the cadaveric setting with mean deviations from the planned version and inclination of around 2° to 3°. These were good results compared to other currently available techniques for glenoid baseplate placement. A study by Throckmorton et al. [38] showed that glenoid components placed with patient-specific guides averaged 5° of deviation from the intended position in version and 3° in inclination; those with standard instrumentation averaged 8° of deviation in version and 7° in inclination.

Equally important, immersive technologies have been shown to reduce inter-surgeon variability, with junior and senior surgeons achieving comparable implant accuracy, as shown by Dey Hazra et al. [29]. Such findings suggest that immersive systems may not only improve outcomes but also flatten the learning curve in shoulder arthroplasty, a feature of particular relevance in training centers or high-turnover clinical settings.

Three further studies transferred the new technology into the operating room, including the two studies by Gregory and Kopriva [22,23], who used immersive technology to visualize the surgical plan. The study by Gregory et al. was conducted in a multicenter setting to perform some of the cases together with international colleagues. This might be of special relevance in complicated cases and also for teaching and demonstration purposes.

Interestingly, only the study by Rojas et al. [24] used AR for surgical navigation, including the tracking of the glenoid with a special marker and positioning of a reference marker at the coracoid process. This study is the current hallmark of the AR application, showing a deviation from the entry point of 2.0 mm and a deviation from the planned version and inclination of 3.4° and 2.5°, respectively, which proved transferability of the cadaveric results to the operating room.

Interestingly, none of those studies has applied the immersive technology to optimize screw positioning, which is also of great importance, especially in dysplastic glenoids with compromised bone stock. The NextAr system (Medacta) that was used in Rojas’s study [24] should be able to navigate screw positioning, but it was not further analyzed by the authors. Another shortcoming of most of the included trials was the omission of medialization and lateralization of the base plate, which are standard features in robotic systems [39,40] and common GPS navigation technologies [41,42]. Again, the work of Rojas et al. [24] was one of the few looking at this particular outcome. Further, navigation of stem implantation was not part of any of the studies. Nevertheless, the inclusion of those features might only be a matter of time.

Some further limitations with special attention to the study designs have to be mentioned. Most of the data to date remain confined to single-center studies, cadaveric validation, or retrospective case series. The lack of randomized controlled trials with long-term clinical follow-up limits conclusions regarding patient-centered outcomes, such as implant longevity or functional recovery. Additionally, heterogeneity in navigation platforms makes it difficult to generalize findings across systems.

### 4.3. General Outlook

The general outlook on those immersive technologies is, in theory, very promising. However, despite promising accuracy and precision outcomes, limitations in usability, like headset fatigue among surgeons using head-mounted displays, and implementation are still unclear. Institutional commitment is required to implement new techniques, with team training required for better integration into sterile surgical workflows and adapting to quicker usage. Further, the costs of implementing those techniques also remain unknown. One very interesting development was that Microsoft officially announced to stop the support for HoloLens 2 with no further versions of the product [43]. Among the large manufacturers of RTSA designs, Stryker, Zimmer Biomet, and Arthrex relied on their product development strategy for that device, and the near future is uncertain. Contrarily, the only system with a clinical study was NextAR from Medacta, which relies on the Vuzix Blade smart glasses.

Future research should prioritize clinical studies, optimally in a multi-institutional prospective setting with standardized outcome measures and sufficient follow-up periods. Integration of real-time navigation data into registries, as well as cost-effectiveness modeling, will also be important to guide policy-level adoption and development of improved software and hardware. As hardware and software mature, ensuring compatibility across implant vendors and ease of use across diverse surgical teams will determine the broader clinical impact of these tools.

Another advantage of immersive technologies is that the surgeon leaves the operating room with precise knowledge of the implant’s exact position. This is particularly important in the current debate, looking intensively at component position. Nowadays, preoperative 3D planning software is commonly used to achieve optimal implant position and range of motion. However, most of them do not know if they have ever reached that planned target. To reach the planned target becomes more likely with the use of patient-specific instruments, either 3D printed or reusable. However, if the surgeon does not place the guide correctly, the achieved result might be incorrect. This is much more foreseeable with the application of GPS navigation [41,42] or robotic-assisted RTSA placement [39,40]. However, those systems are expensive and sometimes difficult to implement. Therefore, the immersive technologies mentioned may represent a feasible alternative. However, a major drawback remains the reliance on surface detection using a tracker, as well as the need to position a fixed marker (i.e., at the coracoid process). Most surgeons hesitate to use systems where they would need to manually outline the surface of the glenoid. For that reason, future research must spend more effort on marker-less registration and tracking, which is still difficult to achieve due to the complex presentation of soft tissues and fluids like blood in the real surgical environment.

Apart from that, the implementation of VR in postoperative rehabilitation is another topic potentially worth investigating. The authors found only one study partially meeting the inclusion criteria. Nam et al. [44] investigated VR in postoperative rehabilitation after shoulder surgery; however, only with indirect relevance to RTSA. The group reported on the development and preliminary clinical application of a VR-based rehabilitation program using animations that depict customized home exercises adjusted to the patient’s postoperative week, as demonstrated by an avatar and seen in a headset. The intervention targeted patients recovering from a broad range of shoulder surgeries, although data specific to RTSA patients was not isolated. The authors suggested that immersive VR may offer a promising tool for scalable, home-based rehabilitation. Carnevale et al. [45] evaluated a sensor-based VR system for home-based shoulder rehabilitation. The system used wearable motion trackers to measure translational and rotational displacements. The authors described it as a promising VR tool for monitoring shoulder kinematics during rehabilitation, making it a viable alternative to traditional motion analysis systems. This study, however, included only healthy volunteers. Due to the absence of RTSA-specific data, this represents an important gap in the current literature and highlights the possible need for prospective trials specifically targeting this population.

## 5. Conclusions

In summary, current studies collectively support the application of immersive navigation technologies in improving implant positioning, which might potentially enhance surgical outcomes. While technical limitations remain, the current evidence base supports their continued development, evaluation, and selective implementation in both training and clinical practice.

## Figures and Tables

**Table 1:** Educational studies.

First Author	Year	Study Type	Participants	Outcomes	Key Findings
Lohre [17]	2020	RCT	Orthopedic residents	OSATS scores, task time	VR group had faster times for completion of cadaveric procedure (*p* = 0.13), higher cumulative OSATS scores (*p* < 0.001)
Lohre [18]	2020	RCT	Senior orthopedic residents and consultants	OSATS scores, task time	Higher OSATS scores for instrument handling (*p* = 0.03), faster task time (*p* = 0.04)
Crockatt [19]	2023	RCT	Junior orthopedic residents	OSATS, GRS, task time	No significant differences; VR non-inferior to cadaver labs
Erickson [20]	2024	RCT	Junior orthopedic residents	Entry point, inclination, version	Improved guidewire placement in dysplastic glenoids
Bischofreiter [21]	2025	Retrospective case series	Single High-volume shoulder surgeon	Surgical time, blood loss	Surgical time reduced (*p* < 0.001), blood loss reduced (*p* = 0.005) along learning curve

Overview of studies for educational and teaching purposes.

**Table 2:** Preclinical trials.

First Author	Year	Study Type	Participants	Technology	Evaluated Items
Berhouet [30]	2017	Feasibility	Printed scapula model	Epson Moverio BT-200 smart glasses	First implementation to superimpose a 3D model
Kriechling [31]	2020	Feasibility	10 3D-printed scapula models	MS HoloLens 1 AR-based guidance	Entry point, angular deviation
Schlueter-Brust [32]	2021	Feasibility	9 3D-printed scapula models	MS HoloLens 1 AR-based guidance	Entry point, angular deviation
Abdic [37]	2023	Feasibility, comparative	128 3D-printed deformed scapula models	MS HoloLens 2 AR-based guidance vs. 3D software planning	Entry point, version, inclination, procedural time, surgeon’s confidence
Gu [35]	2023	Feasibility	30 3D-printed scapula models	MS HoloLens 2 AR-based guidance	Entry point, angular deviation
Trehin [36]	2023	Feasibility	13 3D-printed scapula models	MS HoloLens 2 AR-based guidance	Entry point antero-inferior and supero-inferior, inclination, version, global vector, rotation, procedural time
Fleet [33]	2024	Feasibility, comparative	20 3D-printed scapula models	Traditional vs. PSI vs. MR Stryker navigation	Entry point, version, inclination
Italia [34]	2024	Feasibility, comparative	60 3D-printed scapula models	Traditional vs. MR overlay vs. MR navigation	Entry point, version, inclination, outliers
Kriechling [25]	2021	Cadaveric	12 Cadaver models	MS HoloLens AR-based guidance	Entry point, angular deviation
Rojas [26]	2023	Cadaveric	12 Cadaver models	NextAR HMD	Entry point, version, inclination, rotation, depth, procedural time, outliers, complications
Sanchez-Sotelo [27]	2024	Cadaveric, 7 surgeons	14 Cadaver models	Stryker, MS HoloLens 2	Entry point, version, inclination, superior-inferior, anterior-inferior position, complications
Dordain [28]	2025	Cadaveric	10 Cadaver models	Pixee AR-HMD	Entry point as superior-inferior and anterior-posterior, version, inclination, outliers
Dey Hazra [29]	2025	Cadaveric	16 Cadaver models	NextAR HMD	Version, Inclination, position anterior-posterior, superior-inferior, lateral-medial

Overview of the included studies in a preclinical setting. Abbreviations: HDM—Head-mounted display.

**Table 3:** Clinical studies.

Author	Year	Study Type	n	Technology Application	Evaluated Items
Gregory [22]	2022	Multicenter feasibility	13	Visualization, HoloLens for pre-op Plan	Satisfaction of surgeons
Kopriva [23]	2024	Retrospective comparative	25/72	Stryker Holoblueprint Visualization, no navigation	Version, Inclination, Outliers, Surgical time
Rojas [24]	2025	Prospective multicenter study	17	NextAR HMD Navigation	Entry point, version, inclination, rotation, depth, procedural time, outliers, complications, blood loss

Overview of clinical studies with application of augmented reality in the operating room. Abbreviations: HMD—Head-mounted display.

**Table 4:** Detailed analysis of all parameters.

Study	Entry (mm)	Version (°)	Inclination (°)	Outliers
Kriechling [31]	2.3 ± 1.1	2.7 ± 1.3		-
Schlueter-Brust [32]	2.4 ± 0.7	3.9 ± 2.4		-
Abdic [37]	2.1 ± 0.1	9 ± 1	8 ± 1	
Gu [35]	1.5 ± 1.0	2.4 ± 0.9		
Trehin [36]	0.5 ± 0.4 (vert.) 0.8 ± 0.6 (horiz.)	0.97 ± 0.8	0.89 ± 0.6	-
Fleet [33]	2 ± 1	1 ± 1	2 ± 1	-
Italia [34]	3.3 ± 2.0	4 ± 3	7 ± 5	1
Kriechling [25]	3.5 ± 1.7	3.8 ± 1.7		-
Rojas [26]	1.1 ± 0.4	1.8 ± 1.3	1.0 ± 0.7	0/10
Sanchez-Sotelo [27]	1.7 ± 0.8	1.6 ± 1.2	1.7 ± 1.5	-
Dordain [28]	1.1 ± 1.7 (vert.) 0.5 ± 0.9 (horiz.)	0.7 ± 0.5	0.9 ± 1.6	1/10
Dey Hazra [29]	-	-	3 ± 2	0/12
Gregory [22]	-	-	-	-
Kopriva [23]	-	2.3 ± 2.1	2.4 ± 1.7	1/25
Rojas [24]	2.0 ± 2.5	3.4 ± 4.6	2.5 ± 3.2	3/17

Detailed analysis of the most important measurements: Entry-point (mm), version (°), and inclination (°) deviation from the plan.
